# Using Active Remote Sensing to Evaluate Cloud-Climate Feedbacks: a Review and a Look to the Future

**DOI:** 10.1007/s40641-017-0067-9

**Published:** 2017-05-29

**Authors:** Gerald G. Mace, Elizabeth Berry

**Affiliations:** Department of Atmospheric Sciences, University of Utah, 135 South 1460 East Rm 819 (WBB), Salt Lake City, UT 84112-0110 USA

**Keywords:** Cloud-climate feedbacks, Active remote sensing, Cirrus clouds, Boundary layer clouds, Cloud microphysics

## Abstract

Uncertainty in the equilibrium climate sensitivity (ECS) of the Earth continues to be large. Aspects of the cloud feedback problem have been identified as fundamental to the uncertainty in ECS. Recent analyses have shown that changes to cloud forcing with climate change can be decomposed into contributions from changes in cloud occurrence that are proportional to globally averaged temperature change and changes associated with rapid adjustments in the system that are independent of changes to globally averaged surface temperature. Together these responses enhance warming due to (1) cloud feedback from increasing cloud altitude by upper tropospheric clouds and (2) decreases in cloud coverage by marine boundary layer clouds. We argue that active remote sensing from space can play a unique and crucial role in constraining our understanding of these separate phenomena. For 1, the feedback associated with changing tropical cirrus is predicted to emerge from the statistical noise of the climate system within the next one to two decades. However, active remote sensing will need to continue for that signal to be observed since accurate placement of these clouds in the vertical dimension is necessary. For 2, the processes associated with changes to marine boundary layer clouds have been linked to the coupling between cloud and precipitation microphysics and air motions over remote ocean basins where precipitation formation in shallow convection is modulated by changes to aerosols and thermodynamics. Exploiting the synergy in combined active and passive remote sensing is likely one of the only ways of constraining our evolving theoretical understanding of low-level cloud processes as represented in cloud-resolving models and for validating global-scale models.

## Introduction

It has been known since the era of the Earth Radiation Budget Satellites in the 1980s [1] that clouds tend to cool the Earth by approximately 20 W m^−2^. In other words, the cloud radiative effect or forcing is *R*_cld_ = *R* − *R* ^0^ ≈  − 20 W m^−2^ where *R* is the temporally and globally averaged net radiation at the top of the atmosphere and superscript 0 denotes clear sky. This tendency to cool the Earth system is about five times the magnitude and of opposite sign to the external radiative perturbation (d*R*_ext_) due to a doubling of CO_2_. Assuming that the climate system is represented by changes to the globally averaged surface temperature ($\delta \overset{-}{T_{\mathrm{sfc}}}$), the change in top of atmosphere net radiative flux (d*R*), associated with changes in the climate system, is defined as a feedback [*λ*, W m^−2^ K^−1^]. A thorough discussion of the primary feedbacks in the climate system is given in [2], and a review of cloud feedbacks (*λ*_cld_) is given in [3]. Uncertainty in the equilibrium climate sensitivity (ECS) defined as $\overset{-}{{\delta T}_{\mathrm{sfc}}}$ due to CO_2_ doubling has been persistently large due primarily to uncertainties in *λ*_cld_ [4, 5].

Our understanding of feedbacks in the climate system has become much more nuanced in recent years [6–16] with the insights that (1) evaluation of *λ*_cld_ must account for changes in the system not associated with clouds that appear to mask *R*_cld_ [11, 12] and (2) that certain changes in the system that appear as contributing to *λ*_cld_ are actually due to rapid changes in the system that are independent of $\overset{-}{{\delta T}_{\mathrm{sfc}}}$ [6–10] and therefore not associated with *λ*_cld_. Furthermore, the concept that short-term (interannual to decadal) records from the current climate can provide empirical or emergent constraints on climate model simulations [17] has provided a theoretical foundation for examining the existing observational record for physical mechanisms that correlate cloud radiative effects with feedbacks in the climate system [18–24].

In this paper, we consider what aspects of the cloud feedback problem might be accessible to evaluation using space-based active remote sensing. We consider the existing record of active measurements from the A-Train era [25] and what might be possible were that observational record extended and perhaps expanded in the future. To address these issues, we first briefly review our current understanding of the cloud forcing and feedback concepts.

## The Nature of the Cloud Response—Forcing and Feedback

Consider that even if the distribution and properties of clouds in the climate system did not change with increasing greenhouse gasses, cloud radiative forcing, *R*_cld_, as defined earlier would tend to become smaller just because the infrared opacity of the clear sky atmosphere would increase as greenhouse gas concentrations increase. In other words, a non-zero *λ*_cld_would be inferred even if clouds did not change. This highlights the need to carefully interpret changes to *R*_cld_ (d*R*_cld_) in terms of *λ*_cld_. In a paradigm introduced by Soden et al. [12], the change in d*R*_cld_ can be expressed in terms of d*R* adjusted for the effects of changes to the net radiation in the cloud-free sky (d*R* ^0^):

1 $$ \mathrm{d}{R}_{\mathrm{cld}} = \mathrm{d} R-\left({K}_T^0\mathrm{d} T+{K}_w^0\mathrm{d} w+{K}_a^0\mathrm{d} a+{G}^0\right) $$

where the terms in the parentheses represent d*R* ^0^. In Eq. 1, *G* refers to *R*_ext_, and the quantities *K*_*x*_refer to the globally averaged radiative kernels ($\frac{\partial R}{\partial x}$) due to *x* where *x* can be temperature (*T*), water vapor (*w*), and albedo (*a*). *K*_*x*_ are functions of the radiative transfer physics such as the radiative properties of clouds and the atmosphere, and they allow for an intuitive evaluation of the feedbacks [13–15]. Alternatively, d*R* can be expressed in terms of the total sky (clear plus cloudy) changes,

2 $$ {\lambda}_{\mathrm{cld}}\overset{-}{{\delta T}_{\mathrm{sfc}}}=\mathrm{d} R-\left({K}_T\mathrm{d} T+{K}_w\mathrm{d} w+{K}_a\mathrm{d} a+ G\right) $$

where now the terms in the parentheses represent changes to the all-sky net radiation not associated with changes to clouds. Subtracting Eq. 2 from 1, the relationship between *R*_cld_ and *λ*_cld_ then becomes

3 $$ {\delta \overset{-}{T_{\mathrm{sfc}}}\lambda}_{\mathrm{cld}}=\mathrm{d}{R}_{\mathrm{cld}} + M $$

where *M* is the difference of the parenthesized terms in Eqs. 1 and 2 and represents the extent to which clouds mask changes to non-cloud feedback terms. This relationship between the cloud forcing, feedbacks, and cloud masking was first derived in [12]. The important insight is the relationship between d*R*_cld_ and *λ*_cld_. A decrease in cloud cover with warming that decreases *R*_cld_ with all else remaining constant, for instance, would result in a positive shortwave *λ*_cld_ and a negative longwave *λ*_cld_ with the net depending on their relative magnitudes. If the cloud properties remained the same with decreasing cloud cover, d*R*_cld_ would approach zero as *R* approaches *R* ^0^.

It is generally understood when evaluating ECS, for instance, that Eq. 3 is considered between two equilibrium climate states that differ by a doubling in CO_2_. However, it was found that following an instantaneous CO_2_ doubling, some changes in clouds happened very quickly (within days or months of the CO_2_ change) independent of $\delta \overset{-}{T_{\mathrm{sfc}}}$ that ultimately drove a top of atmosphere (TOA) radiative flux change [6–10]. These rapid adjustments (*F*_cld_) were independent of $\delta \overset{-}{T_{\mathrm{sfc}}}$ and therefore independent of *λ*_cld_, such that d*R*_cld_ = *F*_cld_ + ${\delta \overset{-}{T_{\mathrm{sfc}}}\lambda}_{\mathrm{cld}}.$ With this understanding, we would rewrite Eq. 3 to account for the adjustments since the radiative change that defines the feedback response (${\delta \overset{-}{T_{\mathrm{sfc}}}\lambda}_{\mathrm{cld}}$) is due to changes in *R*_cld_ that are not associated with rapid adjustments.

4 $$ {\overset{-}{{\delta T}_{\mathrm{sfc}}}\lambda}_{\mathrm{cld}}=\mathrm{d}{R}_{\mathrm{cld}} - {F}_{\mathrm{cld}}+\left({M}^{\prime}\right) $$

where the prime on *M* indicates that *M* now includes masking adjustments for the rapid response of the climate system to the instantaneous CO_2_ doubling. Equation 4 illustrates the relationship between the cloud feedback, the cloud forcing, the adjustments, and the cloud masking.

In a further innovation, Zelinka et al., [14, 15] introduced an approach for calculating λ_cld_ using a cloud occurrence (*C*) radiative kernel *K*_c_ where the terms in the *K*_c_ matrix would be $\frac{\partial R}{\partial C}$ as a function of the column optical depth (*τ*) and cloud top pressure (*P*_CT_) based on the International Satellite Cloud Climatology Project (ISCCP) [26] convention . Then *λ*_cld_ is expressed in terms of the matrix of *K*_c_ times the change in an occurrence frequency matrix (*δC*) of the various cloud types defined in terms of *P*_CT_ and *τ*, *λ*_cld_ = *K*_c_*($\delta C/\overset{-}{{\delta T}_{\mathrm{sfc}}}\Big)$. Note that *δC* is normalized by the globally averaged temperature change over which the changes in cloud occurrence were determined. In addition, methodologies for decomposing the cloud feedback into contributions from *δC*, from changes to *P*_CT_, and changes to *τ* were introduced [9, 16]. This decomposition effectively separates the occurrence frequencies from cloud properties and separates the cloud properties into important orthogonal and observable macrophysical components. For instance, one would expect that *R*_cld_ for a given type defined by *P*_CT_ and *τ* would be reasonably constant at a given latitude over some averaging period like a month while the distribution of cloudiness type might shift as the climate changes [27, 28].

From an observational perspective, there are several challenges to interpreting Eq. 4. First, the distinction between *F*_cld_ and d*R*_cld_ are pedagogical in a practical sense since the distinction between *F*_cld_ and *δT*_sfc_ *λ*_cld_ are not observable. However, it is important to know which processes that drive observable changes to the system are and are not associated with feedbacks since they can indicate over what timescales and under what conditions certain change-related processes might emerge from the noise of the climate system. Active remote sensors are uniquely able to observe *C* with very high accuracy and relatively little ambiguity. As we discuss in the following, the influence of cloud-type changes within the *δC* matrix have distinct signatures in the climate system. Also, while it is generally assumed that *K*_c_ is more or less constant [9, 14–16], changes to the diurnal distribution of cloud occurrence and changes to radiative properties due to microphysical processes that may be a function of temperature can modulate *K*_c_. In the next section, we elaborate on these issues using specific examples from the measurement record and from recent literature.

## The Role of Active Remote Sensors

Using Eq. 5 as a roadmap, we consider the role of active remote sensing in contributing to our understanding. The challenge that must be overcome is that the conceptual thinking that produced Eq. 5 is rooted in global modeling where equilibrium climate states can be generated numerically from instantaneously perturbed approximations of nature. Because of the drastically different space and timescales of such global modeling exercises compared to measurements and because it is difficult to conduct controlled experiments using the actual Earth, the observational record has played a limited role in developing understanding and in constraining predictions regarding climate feedbacks and forcings. We argue that the primary contribution of active and combined active and passive remote sensors such as the A-Train will be in populating the *C* matrix on global spatial scales and on timescales that are becoming climate-relevant. This is especially true in light of the emergent constraint concept [17]. To illustrate how active remote sensors can contribute to the cloud feedback/forcing problem, we present an example.

### *K*_c_*C* = *R*_cld_ : an Example

**Fig. 1:**
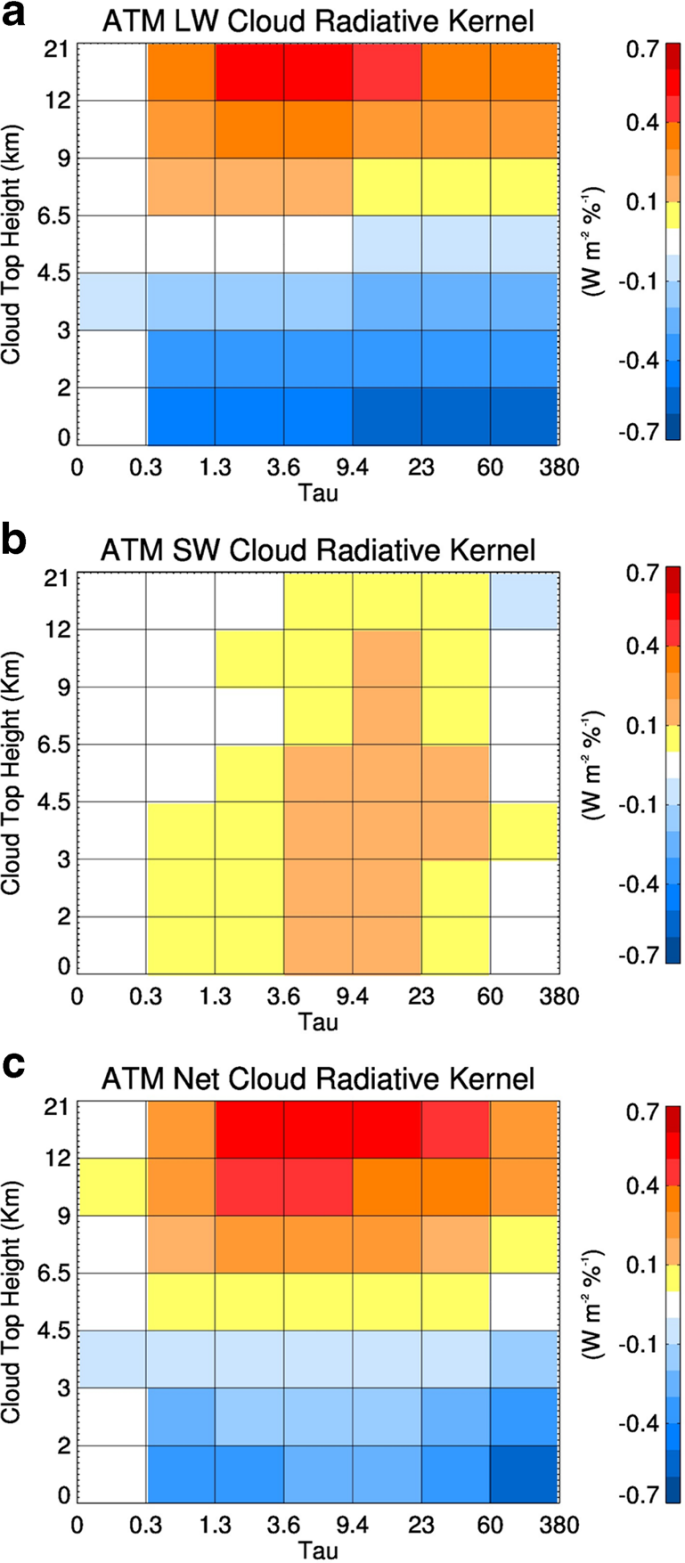
Atmospheric radiative kernels in units of W m^−2^ %^−1^ following the approach by Zelinka [9, 16] calculated from single-layer cloud properties from A-Train data over the North Atlantic [35] between 45° N–65° N and 10° W–30° W during calendar year 1997. **a** In-atmosphere longwave kernel. **b** In-atmosphere shortwave kernel. **c** In-atmosphere net radiative kernel

**Fig. 2:**
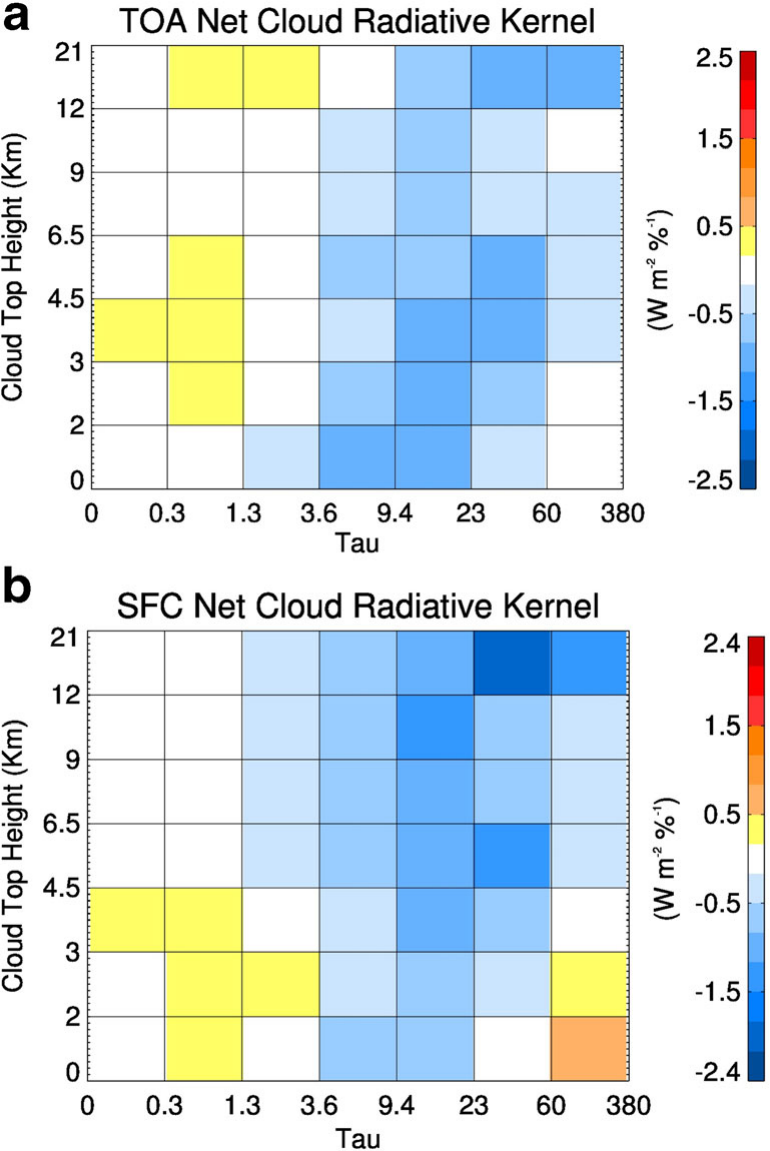
As in Fig. 1 except **a** the TOA net radiative kernel and **b** the surface net radiative kernel

**Fig. 3:**
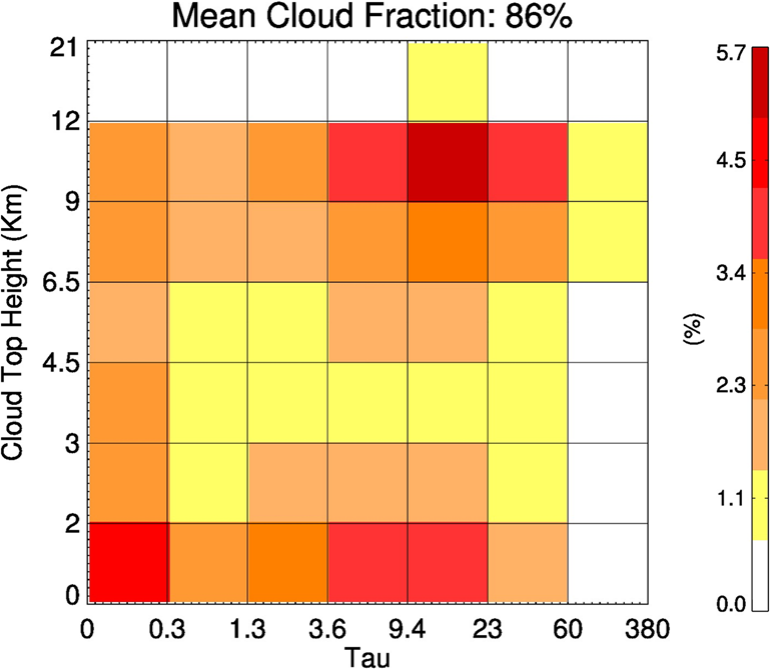
The cloud-type occurrence matrix *C* from the North Atlantic analysis region during 2007 as derived from A-Train active and passive remote sensing data [36]

**Fig. 4:**
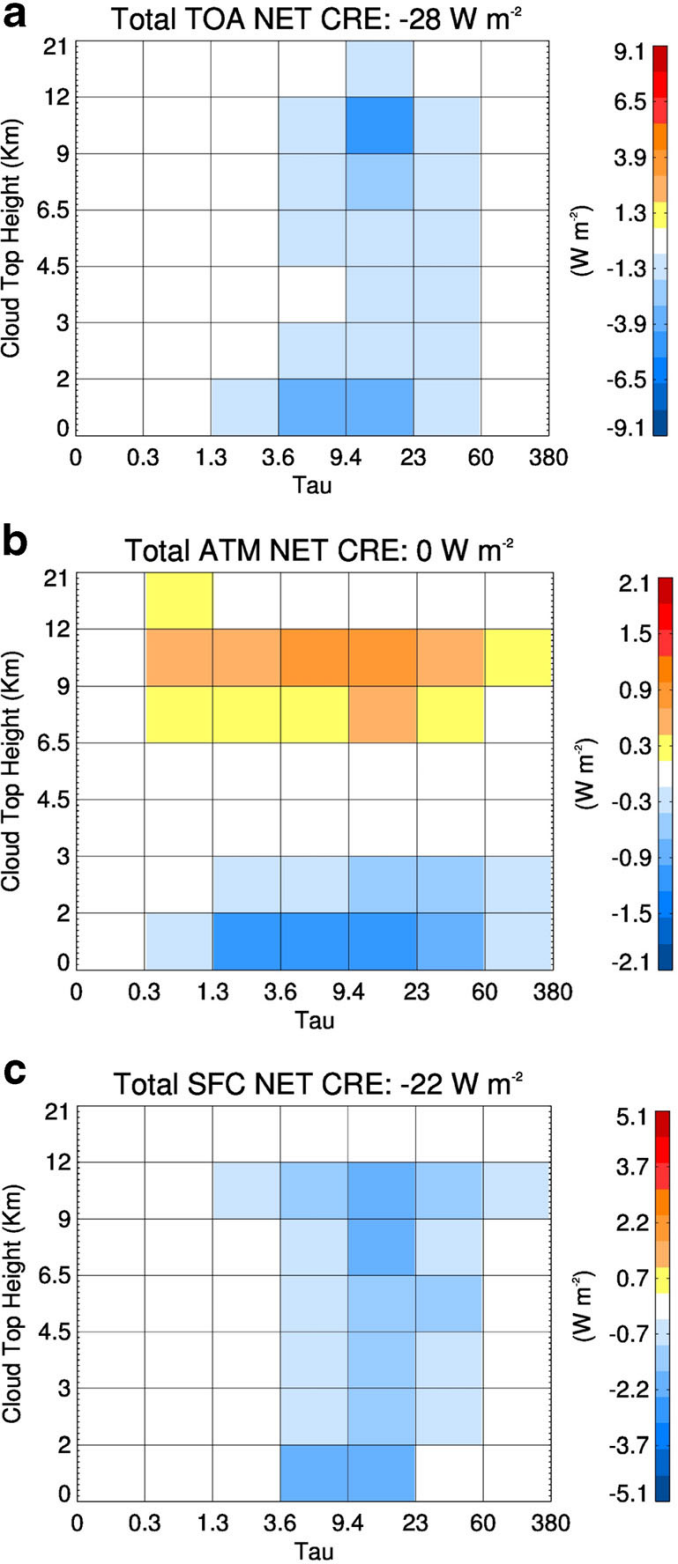
Cloud radiative effect (*R*_cld_) matrix resulting from multiplying the cloud fraction with the TOA (**a**), atmosphere (**b**), and surface (**c**) net cloud radiative kernels, calculated from cloud layer properties derived from A-Train data over the North Atlantic for calendar year 2007 [37]. The sum of each matrix (W m^−2^) is shown in the title. The uncertainty in the net CRE estimates is approximately 7 W m^−2^

Active remote sensors, particularly millimeter wavelength radar and elastic lidar, are uniquely suited to observing the vertical distribution of clouds and precipitation [29, 30] and important first-order properties such as cloud top phase [31–34]. In Figs. 1, 2, 3, and 4, we present an example of using combined space-borne lidar and radar data to diagnose *R*_cld_ in terms of the radiative kernel and occurrence matrix.

Examples of *C*, *K*_c_, and *R*_cld_ matrices were developed following the methodology of [14] using data collected in a 20° × 20° region in the North Atlantic centered roughly on 55° N and 20° W. An entire annual cycle is used in this analysis, and we present an average of day and night measurements. The in-atmosphere *K*_c_ or *K*_c,atm_ in Fig. 1 is an observationally derived radiative kernel that was created using fluxes calculated by combining active and passive A-Train measurements and retrievals as described in detail in [37]. Here, we present *K*_c_ in terms of the geometric cloud top height (CTH) instead of *P*_CT_ to illustrate the advantage provided by active remote sensors in avoiding the inherently ambiguous *P*_CT_ [35, 38]. Unlike the *K*_c_ shown in [9], these matrices are not necessarily smooth since each CTH-τ bin averages flux data from cloud layers with varying microphysical properties (phase, water path, effective radius). In addition, the observationally derived radiative kernel is limited by the sample size in each CTH-τ bin (Fig. 3) and is influenced by the diurnal variation of cloud layers, with some cloud types occurring more frequently during the night overpass and vice versa.

The net *K*_c,atm_ is largely dominated by longwave radiation and demonstrates the bifurcation in upper tropospheric heating and lower tropospheric cooling by clouds over these middle-latitude regions. Conversely, the net TOA and surface *K*_c_ in Fig. 2 are similar to one another, implying that most clouds strongly cool the surface, and this cooling is predominant in the column. Note the difference in the color scale between Figs. 1 and 2.

The cloud occurrence matrix or *C* in Fig. 3 shows that the distribution of cloud layer occurrence in this region is dominated by optically thick high-level clouds associated with fronts and moderately optically thick low-level clouds that have tops below 3 km. However, the extremely high cloud fraction in this region of 86% indicates the predominance of clouds to the radiation budget of the North Atlantic. Multiplying *K*_c,TOA_, *K*_c,atm_, and *K*_c,sfc_ by *C* allows us to derive matrices of cloud radiative forcing, *R*_cld_ (Fig. 4). Interestingly, the net radiative forcing of clouds to the atmosphere of the North Atlantic region balances to be effectively zero over the annual cycle considered. Since the atmosphere cloud radiative effect (CRE) balances, the net surface and TOA CRE are effectively identical. The cooling diagnosed in this region on an annual cycle results from optically thick low-level clouds and frontal clouds that have tops in the upper troposphere.

The nuances provided in this example highlight the unique strength of combined space-borne cloud radar and lidar to the cloud feedback problem using the *K*_c_ kernel approach. Because we can place hydrometeor layers in the atmosphere with very high accuracy using the active remote sensors, we can also derive their radiative properties with improved accuracy. This allows us to demonstrate not only that the atmosphere CRE effectively balances, but that this balance is achieved through cooling in the lower troposphere and heating aloft. Presumably, the energy that is deposited in the upper troposphere is then exported to higher latitudes where it is ultimately radiated to space. It is noteworthy that analyzing only the TOA radiative quantities misses the interesting radiative processes that occur in the atmosphere [37].

### Observations: Practical Questions

While the overall picture of forcing and feedbacks is a complicated one, we make an attempt at extreme simplification. Two primary phenomena associated with temperature-mediated feedbacks and rapid adjustments seem to predominate.

1. High-level clouds ascend in height as the climate warms inducing a positive longwave feedback [39].
2. Marine boundary layer (MBL) clouds decrease in coverage inducing a positive shortwave feedback^1^ [40].

These feedbacks would be realized by changes to the *δC* matrix. Ascending high clouds would be realized by a migration of non-MBL cloud tops (particularly upper tropospheric ice clouds hereafter referred to as cirrus) upward. The MBL cloud feedbacks would be realized as a decrease in overall cloud coverage resulting in more solar radiation absorbed at the surface, while the ascent of high clouds is thought to be understood [39, 41] and is a robust feature of climate predictions [13]. It is important to note that both of these are positive feedbacks, and neither has been observationally verified although the use of interannual variability in the measurements as climate feedback proxies is providing hints that the model predictions are credible [17–20, 42, 43].

With these issues in mind, we argue that solving the cloud feedback problem in general or even the two simplified aspects of the problem listed earlier has two necessary conditions. The first would be understanding. Why is a particular feedback happening? For ascending high clouds, the answer to whether we understand the phenomenon is thought to be yes [39, 41]. All models seem to agree more or less that high clouds will ascend with warming and there are physically plausible explanations for it [13] and measurements are hinting that it is happening [44–47]. For decreasing MBL coverage, it is very clear, given the disparity in ECS that has been linked to decreasing MBL coverage, that we do not understand this phenomenon [4, 36, 40, 42, 43, 48–52] although physically plausible mechanisms have been proposed [36, 48]. A second necessary condition to solving the cloud feedback problem would be observational verification. Have the processes associated with the phenomenon been observed and/or has the feedback itself been observed? More importantly perhaps, at what point do we expect the signal of a particular phenomenon to emerge from the natural noise in the climate system so that observational verification can take place [53, 54]?

### High Clouds

Regarding the ascent of high clouds, we will not elaborate on our first condition for solution (understanding) since a series of papers have documented a plausible theoretical mechanism for it. Our second condition for solution (verification) however raises very interesting questions. The recent report by Norris et al., [55] examines a 30-year record of *P*_CT_-τ *C* from ISCCP and PATMOS-x [56] compared to similarly processed model statistics. They report statistically significant spatial shifts in cloud occurrence as well as changes to the vertical distribution of cloudiness (Fig. 3 of [55]). In particular, they identify an increase in high cloud occurrence that seems to confirm model predictions of similar changes although caution is warranted given the ambiguities associated with the use of passive remote sensing to populate *P*_CT_-τ-dependent *C* histograms [35, 38].

While the TOA radiative signature of climate change is not expected to emerge from natural variability for decades [57, 58], the *δC* for tropical cirrus may be accessible to remote sensing observations in the relatively near future [53, 54]. If model predictions are realistic, the emergence of this feedback signal in observations could occur in the 2020s. Identifying such a signal in measurements (or not) would represent a unique observational constraint on climate models. Regardless, use of the existing observational record is producing intriguing examples of *λ*_cld_derived from CALIPSO data [18–20] using the emergent constraint concept [17] that suggests that this signal indeed may be emerging from climate system noise although caution is warranted [44].

### MBL Clouds

The feedback problem associated with MBL clouds is very nearly the mirror image of the high cloud problem. With models widely disagreeing on the magnitude and even the sign of this feedback (although evidence is suggesting that it is positive), it is clear that we are not close to a sufficient understanding of the physical processes that will drive the climate system one way or another [4, 36, 40, 42, 43, 48–52]. Our second condition for solution—observational verification—seems somewhat out of reach for the cloud coverage problem since the signal of MBL cloud coverage decrease is not expected to emerge from the climate system noise until beyond the 2030s [53, 54] although the existing data record is providing hints about the nature of this feedback mechanism [21–24]. We argue that focusing on understanding the dominant physical processes involved in MBL cloud coverage changes is a critical activity to which active remote sensing from space can uniquely contribute.

A series of competing processes appear to be at work in MBL cloud feedbacks. These processes range from how free tropospheric air is mixed into deeper and warmer boundary layers [36, 48] and how latent heat fluxes required to maintain a constant relative humidity dry the MBL [50, 51] to the fact that moist adiabatic lapse rates become steeper with warming. This latter process results in higher liquid water paths [36] especially at colder temperatures and higher latitudes where cloud phase changes will also vary and additional negative feedbacks could modulate the overall response [21–24]. Other processes are expected to play a role including aerosol-cloud interactions [52]. Few of these processes have specifically been constrained observationally although there is evidence that the overall interannual responses in many climate models are not consistent with bulk cloud properties derived from measurements [21]. While the process of mixing dry air into the MBL seems to be gaining consensus as an explanation for why models produce decreasing low-level clouds, it is important to note that the properties of the free tropospheric air that mixes depends on the degree to which this air has been modified by shallow penetrative convection [36, 49].

The life cycles of these shallow convective clouds are thought to be linked to the aerosol-mediated droplet number via the processes that modulate precipitation formation [36, 50–52]. Quantitative empirical knowledge of these processes is uniquely provided only by simultaneous knowledge of cloud and precipitation properties within turbulent MBLs [59–64]. An observational capacity for diagnosing CTH, cloud droplet number, and associated precipitation microphysics in cloud elements is one necessary aspect to provide an observational constraint on competing feedbacks and processes that may cause changes to boundary layer cloud cover over the coming decades. Diagnosing CTH, cloud droplet number, and precipitation microphysics in cloudy vertical columns is an extremely challenging proposition that cannot be accomplished with any single instrument but requires combinations of passive and active remote sensing consisting minimally of millimeter radar, lidar, solar reflectance, and passive microwave measurements as demonstrated with A-Train data [59–64].

## Summary and Conclusions

In a recent discussion of grand challenges, Bony et al. [49] report that most of the leading advances in our understanding of cloud feedback and climate processes have come primarily from idealized modeling, yet there remain major outstanding uncertainties that must be addressed observationally if we are to reduce the spread in ECS. It is, therefore, incumbent on the observational community to keep pace with the questions being posed by the theoretical community. Because it typically takes a decade or more to move a new observational platform or concept to space, the observational community must be thinking well ahead.

The real strength of the A-Train has been, first and foremost, to accurately and unambiguously map the vertical distributions of hydrometeor layers in the atmosphere. This accurate mapping relates directly to the occurrence matrix, *C*, alluded to in “The Nature of the Cloud Response—Forcing and Feedback” section and illustrated in Fig. 3. Additionally, the radar and lidar in the A-train illustrate how active remote sensing characterizes important moments of the vertical hydrometeor distributions that can be combined synergistically with passive measurements to provide integral constraints on the column properties [65, 66]. With more than a decade of A-Train data, opportunities exist for innovative exploration of this data to increase our understanding of cloud and precipitation processes and properties within the general circulation. This is especially true with the evolving concept of using the interannual measurement record to constrain the cloud responses in climate models [17].

With the A-Train era ending late this decade or earlier as the active instruments reach end of life, we have argued that there is very good reason to continue a constellation-based active and passive measurement strategy. Models predict that *δC* signals in cirrus may be identifiable from the natural noise in the system sometime in the third decade of this century [53, 54] and thereby provide a unique observational constraint on GCMs and our understanding of the Earth’s climate. Beyond this, the cloud feedback decomposition into *K*_c_ and *C* allows us to examine cloud-circulation coupling using longer data records. Furthermore, with the technological advances of the last decade [67–73] and our increasing understanding of how to exploit multi-instrument, multi-frequency active and passive synergy that began with the A-Train, the observational community is well positioned to address the aerosol-cloud-precipitation processes that presently drive the large uncertainty in ECS.

## Footnotes

1 Note that the decrease in low-level clouds has also been linked to rapid adjustments [9], but from a practical observational aspect, we combine those effects here into a single climate system response. The MBL positive feedback is modulated by negative feedbacks in the system such as what is known as the optical depth feedback that occurs primarily at colder temperatures [21].

## References

[1] Ramanathan V (1987). The role of earth radiation budget studies in climate and general circulation research. J Geophys Research.

[2] Soden BJ, Held IM (2006). An assessment of climate feedbacks in coupled ocean-atmosphere models. J Clim.

[3] Stephens GL (2005). Cloud feedbacks in the climate system: a critical review. J Atmos Sci.

[4] Dufresne J-L, Bony S (2008). An assessment of the primary sources of spread of global warming estimates from coupled atmosphere-ocean models. J Clim.

[5] Stevens B, Bony S (2013). What are climate models missing?. Science.

[6] Andrews T, Forster PM. CO2 forcing induces semi-direct effects with consequences for climate feedback interpretations. Geophys Res Letters. 2008;35 doi:10.1029/2007GL032273.

[7] Gregory JM, Webb M (2008). Tropospheric adjustment induces a cloud component in CO2 forcing. J Clim.

[8] Sherwood SC, Bony S, Boucher O, Bretherton C, Forster PM, Gregory JM, Stevens B. Adjustments in the forcing-feedback framework for understanding climate change. Bull Am Meteorol Soc. 2013; doi:10.1175/BAMS-D-13-00167.1.

[9] Zelinka MD, Klein SA, Taylor KE, Andrews T, Webb MJ, Gregory JM, Forster PM (2013). Contributions of different cloud types to feedbacks and rapid adjustments in CMIP5. J Clim.

[10] Andrews T, Gregory JM, Forster PM, Webb MJ (2011). Cloud adjustment and its role in CO2 radiative forcing and climate sensitivity: a review. Surv Geophys.

[11] Soden GJ, Broccoli AJ, Hemler RS (2004). On the use of cloud forcing to estimate cloud feedback. J Clim.

[12] Soden BJ, Held IM, Colman R, Shell KM, Kiehl JT, Shields CA (2008). Quantifying climate feedbacks using radiative kernels. J Clim.

[13] Soden BJ, Vecchi GA (2011). The vertical distribution of cloud feedback in coupled ocean-atmosphere models. Geophys Res Lett.

[14] Zelinka MD, Klein SA, Hartmann DL (2012). Computing and partitioning cloud feedbacks using cloud property histograms. Part I: Cloud radiative kernels, Journal of Climate.

[15] Zelinka MD, Klein SA, Hartmann DL (2012). Computing and partitioning cloud feedbacks using cloud property histograms. Part II: attribution to changes in cloud amount, altitude, and optical depth, Journal of Climate.

[16] Zelinka MD, Zhou C, Klein SA. Insights from a refined decomposition of cloud feedbacks. Geophys Res Lett. 2016;43 doi:10.1002/2016GL069917.

[17] Klein SA, Hall A. Emergent constraints for cloud feedbacks. Curr Clim Change Rep DOI. 2015; doi:10.1007/s40641-015-0027-1.

[18] Dessler AE (2010). A determination of the cloud feedback from climate variations over the past decade. Science.

[19] Zhou C, Zelinka MD, Dessler AE, Klein SA (2015). The relationship between interannnual and long-term cloud feedbacks. Geophys. Res. Letters.

[20] Zhou C, Dessler AE, Zelinka MD, Yang P, Wang T (2014). Cirrus feedback on interannual climate fluctuations. Geophys Res Letters.

[21] Gordon ND, Klein SA. Low-cloud optical depth feedback in climate models. J Geophys Res Atmos. 119:6052–65. doi:10.1002/2013JD021052.

[22] Ceppi P, Hartmann DL, Webb MJ (2016). Mechanisms of the negative shortwave cloud feedback in middle to high latitudes. J Clim.

[23] Ceppi P, Mccoy DT, Hartmann DL (2016). Observational evidence for a negative shortwave cloud feedback in middle to high latitudes. Geophys Res Lett.

[24] Terai CR, Zelinka M, Klein SA (2016). Constraining the low cloud optical depth feedback at middle and high latitudes using satellite observations. J Geophys Res Atmos.

[25] Stephens GL (2008). CloudSat mission: performance and early science after the first year of operation. J Geophys Res.

[26] Rossow WB, Schiffer RA (1999). Advances in understanding clouds from ISCCP. Bull Amer Meteor Soc.

[27] Butler AH, Thompson DW, Heikes R (2010). The steady-state atmospheric circulation response to climate change-like thermal forcings in a simple general circulation model. J Clim.

[28] Simpson IRT, Shaw A, Seager R (2014). Diagnosis of the seasonally and longitudinally varying midlatitude circulation response to global warming. J Atmos Sci.

[29] Mace GG, Zhang Q, Vaughn M, Marchand R, Stephens G, Trepte C, Winker D (2009). A description of hydrometeor layer occurrence statistics derived from the first year of merged Cloudsat and CALIPSO data. J Geophys Res.

[30] Mace GG, Zhang Q. The Cloudsat radar-lidar geometrical profile algorithm (RL-GeoProf): updates, improvements, and selected results. J Geophys Res. 2014; doi:10.1002/2013JD021374.

[31] Hu Y, Rodier S, Xu K, Sun W, Huang J, Lin B, Zhai P, Josset D (2010). Occurrence, liquid water content, and fraction of supercooled water clouds from combined CALIOP/IIR/MODIS measurements. J Geophys Res.

[32] Tan I, Storelvmo T, Zelinka MD (2016). Observational constraints on mixed-phase clouds imply higher climate sensitivity. Science.

[33] Kay JE, Bourdages L, Miller NB, Morrison A, Yettella V, Chepfer H, Eaton B (2016). Evaluating and improving cloud phase in the Community Atmosphere Model version 5 using spaceborne lidar observations. J Geophys Res Atmos.

[34] Kay JE, Wall CJ, Yettella V, Medeiros B, Hannay C, Caldwell P, Bitz C. Global climate impacts of fixing the Southern Ocean shortwave radiation bias in the Community Earth System Model. J Clim. 2016; doi:10.1175/JCI-D-15-0358.1. **in press**

[35] Mace GG, Houser S, Benson S, Klein SA, Min Q. Critical evaluation of the ISCCP simulator using ground-based remote sensing data. J Clim. 2010; doi:10.1175/2010JCLI3517.1.

[36] Rieck M, Nuijens L, Stevens B (2012). Marine boundary layer cloud feedback in a constant relative humidity atmosphere. Journal of Atmospheric Science.

[37] Mace GG. Cloud properties and radiative forcing over the maritime storm tracks of the Southern Ocean and North Atlantic derived from A-Train. J Geophys Res. 2010;115 doi:10.1029/2009JD012517.

[38] Mace GG, Wrenn FJ (2013). Evaluation of hydrometeor layers in the East and West Pacific within ISCCP cloud top pressure-optical depth bins using merged CloudSat and CALIPSO data, 2013. J Clim.

[39] Zelinka MD, Hartmann DL (2010). Why is longwave cloud feedback positive?. J Geophys Res.

[40] Bony S, Dufresne J-L (2005). Marine boundary layer clouds at the heart of tropical cloud feedback uncertainties in climate models. Geophys Res Lett.

[41] Hartmann DL, Larson K (2002). An important constraint on tropical cloud-clmate feedback. Geophys Res Lett.

[42] Clement AC, Burgman R, Norris JR (2009). Observational and model evidence for positive low-level cloud feedback. Science.

[43] Myers TA, Norris JR (2016). Reducing the uncertainty in subtropical cloud feedback. Geophys Res Lett.

[44] Li Y, Yang P, North GR, Dessler A (2012). Test of the fixed anvil temperature hypothesis. J Atmos Sci.

[45] Zelinka MD, Hartmann DL (2011). The observed sensitivity of high clouds to mean surface temperature anomalies in the tropics. J Geophys Res-Atmos.

[46] Eitzen ZA, Xu KM, Wong T (2009). Cloud and radiative characteristics of tropical deep convective systems in extended cloud objects from CERES observations. J Clim.

[47] Xu KM, Wong T, Wielicki BA, Parker L, Lin B, Eitzen ZA, Branson M (2007). Statistical analyses of satellite cloud object data from CERES. Part II: tropical convective cloud objects during 1998 El Nino and evidence for supporting the fixed anvil temperature hypothesis. J Clim.

[48] Sherwood S, Sandrine Bony C, Dufresne J-L (2014). Spread in model climate sensitivity to atmospheric convective mixing. Nature.

[49] Bony S, Stevens B, Frierson D, Jakob C, Kageyama M, Pincus R, Shepherd T, Sherwood S, Siebesma AP, Sobel AH, Watanabe M, Webb M (2015). Clouds, circulation, and climate sensitivity. Nat Geosci.

[50] Xu K, Cheng MA, Zhang M (2010). Cloud-resolving simulation of low-cloud feedback to an increase in sea surface temperature. J Atmos Sci.

[51] Bretherton, C. S., P. N. Blossey, and C. R. Jones, Mechanisms of marine low cloud sensitivity to idealized climate perturbations: a single-LES exploration extending the CGILS cases.

[52] Stevens B, Feingold G (2009). Untangling aerosol effects on clouds and precipitation in a buffered system. Nature.

[53] Chepfer H, Noel V, Winker D, Chiriaco M (2014). Where and when will we observe cloud changes due to climate warming?. Geophys Res Lett.

[54] Marvel K, Zelinka M, Klein SA, Bonfils C, Caldwell P, Doutriaux C, Sandter BD, Taylor KE (2015). External influence on modeled and observed cloud trends. J Clim.

[55] Norris JR, Allen RJ, Evan AT, Zelinka MD, O’Dell CW, Klein SA. Evidence for climate change in the satellite cloud record. Nature. 2016; doi:10.1038/nature18273. 10.1038/nature18273 27398619

[56] Heidinger A, Foster K, Walther MJ, Zhao Z (2014). The pathfinder atmospheres Extended (PATMOS-x) AVHRR climate data set. Bull Am Meteorol Soc.

[57] Wielicki BA (2013). Achieving climate change absolute accuracy in orbit. Bull Am Meteorol Soc.

[58] Loeb NG, Kato S, Su W, Wong T, Rose FG, Doelling DR, Norris JR, Huang X. Advances in understanding top of the atmosphere radiation variability from satellite observations. Surv Geophys. 2013; doi:10.1007/s10712-012-9175-1.

[59] Lebsock MD, L’Ecuyer TS (2011). The retrieval of warm rain from CloudSat. J Geophys Res.

[60] Mace GG, Avey S, Cooper S, Lebsock M, Tanelli S, Dobrowalski G. Retrieving co-occurring cloud and precipitation properties of warm marine boundary layer clouds with A-Train data. J Geophys Res. 2016;121 doi:10.1002/2015JD023681.

[61] Mace GG, Avey S. Seasonal variability of warm boundary layer cloud and precipitation properties in the Southern Ocean as diagnosed from A-Train data. J Geophys Res Atmos. 2016;121 doi:10.1002/2016JD025348.

[62] Suzuki K, Stephens GL, van den Heever SC, Nakajima T (2011). Diagnosis of the warm rain process in cloud resolving models using joint CloudSat, MODIS observations. Journal of Atmospheric Science.

[63] Suzuki K, Stephens GL, Lebsock MD (2013). Aerosol effect on the warm rain formation process: satellite observations and modeling. J Geophys Res.

[64] L’Ecuyer TS, Berg W, Haynes J, Lebsock M, Takemura T. Global observations of aerosol impacts on precipitation initiation in warm-topped maritime clouds. J Geophys Res. 2009;114 doi:10.1029/2008JD011273.

[65] Nakajima T, King MD (1990). Determination of the optical thickenss and effective particle radius of clouds from reflected solar radiation measurements. Part I: theory Journal of the Atmospheric Science.

[66] O’Dell CW, Wentz FJ, Bennartz R (2008). Cloud liquid water path from satellite-based passive microwave observations: a new climatology over the global oceans. J Clim.

[67] Müller D, Co-Authors (2014). Airborne multiwavelength high spectral resolution lidar (HSRL-2) observations during TCAP 2012: vertical profiles of optical and microphysical properties of a smoke/urban haze plume over the northeastern coast of the US. Atmos Meas Tech.

[68] Churnside JH. Review of profiling oceanographic lidar. Opt Eng. 2014;53(5):051405–5.

[69] Durden SL, Tanelli S, Epp L, Janmejad V, Long E, Perez R, Prata A (2016). System design and subsystem technology for a future spaceborne cloud radar. IEEE Geosci Remote Sens Lett.

[70] T. Hand, M. Cooley, G. Kempic, D. Sall, P. Stenger, S. Woodworth, R. Park, P. E. Racette, G. Heymsfield, L. Li, 2013: Dual-band shared aperture reflector/reflectarray antenna designs, technologies and demonstrations for NASA’s ACE Radar. *2013 I.E. International Symposium on Phased Array Systems & Technology*, Oct 2013.

[71] Sadowy GA, Sanchez-Barbetty M, Tanelli S, Cannon B, Vanhille K, Brown A, Brown K (2016). Development of a three-frequency spaceborne radar for cloud and precipitation measurement.

[72] S. Tanelli, G.M. Heymsfield, G.S. Stephens, S.L. Durden, E. Im, P. Racette, G.A. Sadowy and L. Li, 2010: Decadal survey tier 2 mission study: summative progress report: ACE Radar. http://ntrs.nasa.gov/search.jsp?R=20120004215

[73] Peral E, Tanelli S, Haddad Z, Sy O, Stephens G, Im E (2015). Raincube: a proposed constellation of precipitation profiling radars in CubeSat.

